# Supplementary material for: Genome-wide identification of natural RNA aptamers in prokaryotes and eukaryotes
Source: Nat Commun. 2018 Mar 29;9:1289. doi: 10.1038/s41467-018-03675-1 (PMC5876405; doi:10.1038/s41467-018-03675-1)
Supplement: Supplementary file 1 — Supplementary information(PDF 12834 kb) [file 41467_2018_3675_MOESM1_ESM.pdf]

Supplementary Information

Genome-wide identification of natural RNA aptamers in prokaryotes and eukaryotes.

Tapsin S. et al.

The supplementary information consists of 12 Supplementary Figures, 3 Supplementary Tables, and Supplementary references.

Supplementary Figure 1

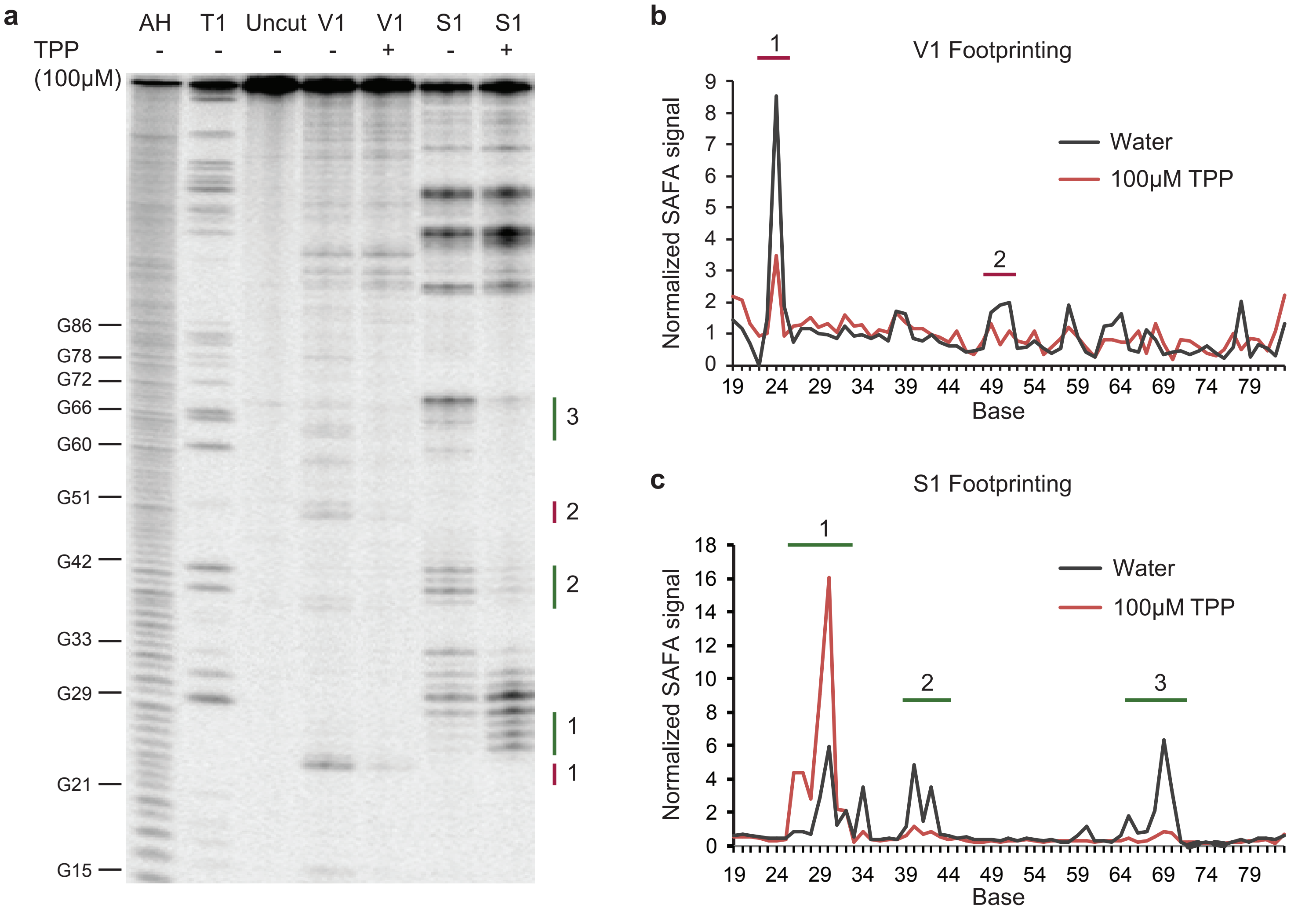

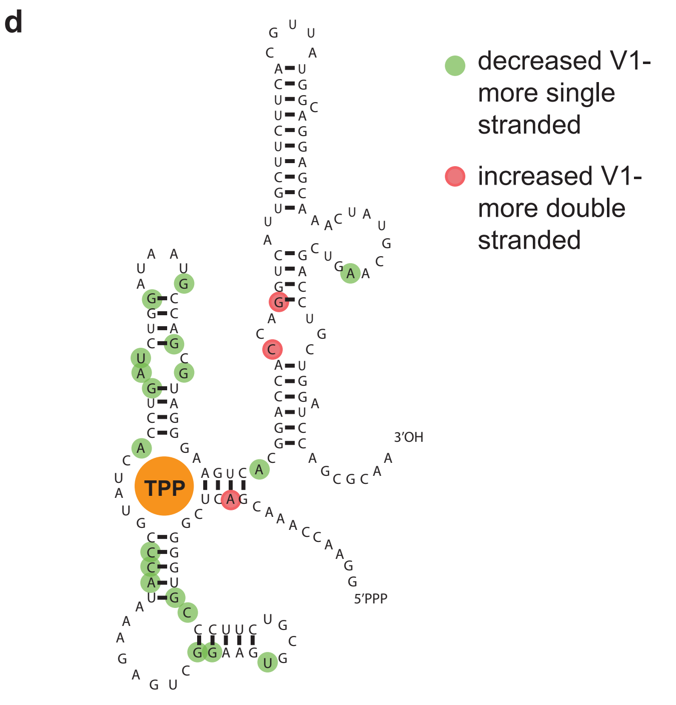

Supplementary Figure 1. PARCEL detects ligand-induced structure changes similar to RNA footprinting. **a**, Gel image of the *E. coli* TPP riboswitch, thiM, structure probed in the presence and absence of 100µM TPP using RNase V1 (lanes 4,5) and S1 nuclease (lanes 6,7). We also include alkaline hydrolysis (lane 1, AH), G ladder (lane 2, T1), and full length RNA without nuclease treatment (lane 3, Uncut) for comparison. The red and green bars indicate double- and single-stranded changing regions respectively. **b,c**, SAFA[1] quantification of the TPP riboswitch structure probed with RNase V1 (**b**) or S1 nuclease (**c**) in the presence (red line) or absence (black line) of TPP. Manual structure probing agrees with high-throughput sequencing data in **Figure 1b**. **d**, Secondary structure model of the TPP riboswitch bound to TPP. The green circles indicate bases that become more single-stranded and red circles indicate bases that become more double-stranded upon TPP binding.

Supplementary Figure 2

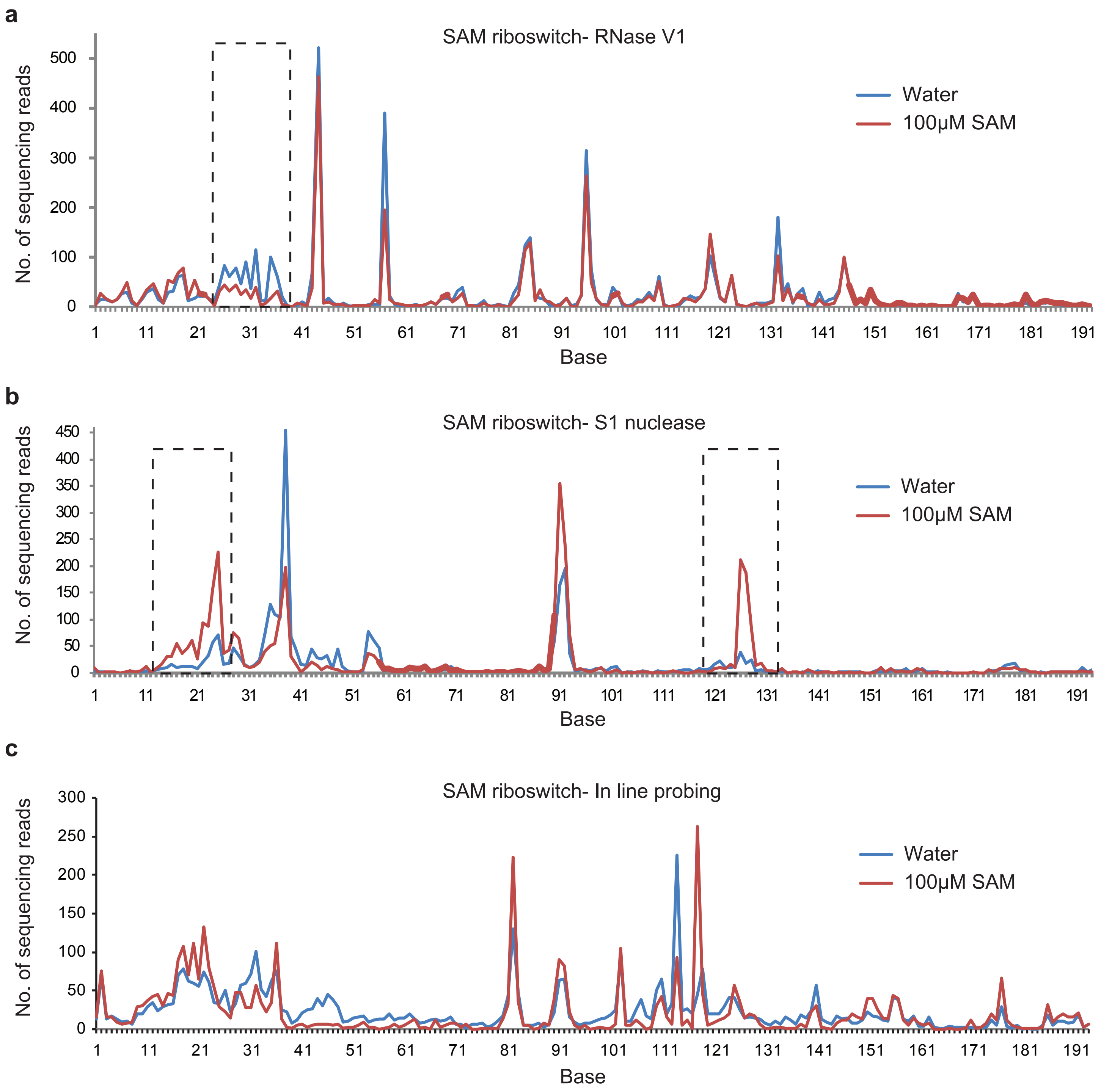

Supplementary Figure 2. PARCEL can detect structure changes for a known SAM riboswitch. High-throughput sequencing reads for the known SAM riboswitch, yitJ gene, in *B. subtilis*[2]. yitJ was structure probed with RNase V1 (**a**), S1 nuclease (**b**), or in-line probing[3] (**c**), in the presence (red line) and absence (blue line) of 100 μM SAM. The dotted box indicates the region of structural change upon ligand binding as detected by deep sequencing.

Supplementary Figure 3

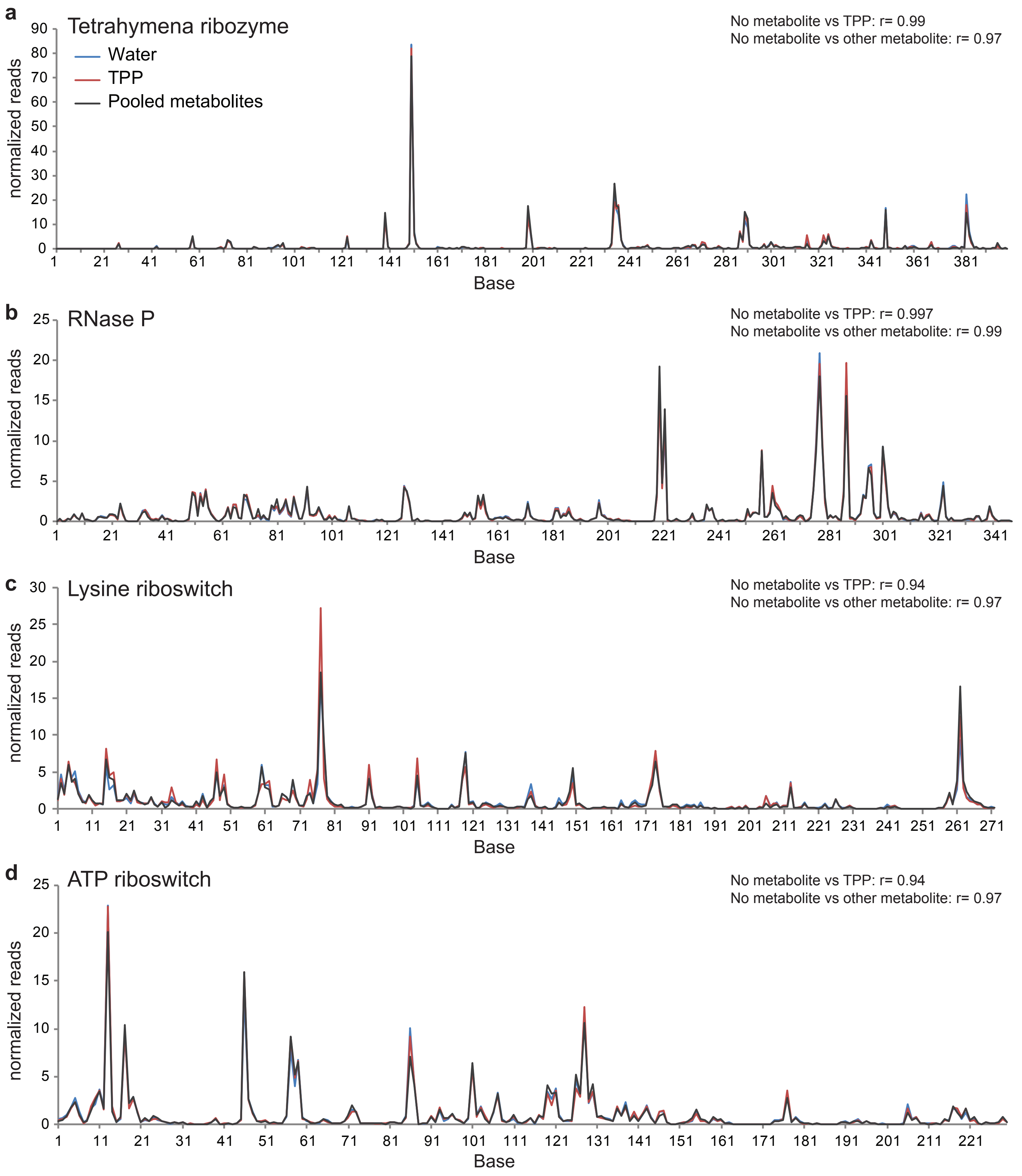

Supplementary Figure 3. PARCEL does not detect structural changes in a set of negative controls. a-d, Sequencing reads for negative controls that are doped into the PARCEL sequencing library. Tetrahymena ribozyme (**a**), *S. cerevisiae* RNase P (**b**), lysine riboswitch[4] (**c**), and ATP riboswitch (**d**) were structure probed with RNase V1 without metabolite (blue line), in the presence of 100 μM TPP (red line) or a pool of five metabolites (SAM, glycine, lysine, FMN and AdoCbl, each 100 μM; black line). These RNAs are not observed to undergo structure changes in the presence of ligands (not known to bind to these RNAs), demonstrating PARCEL's specificity.

Supplementary Figure 4

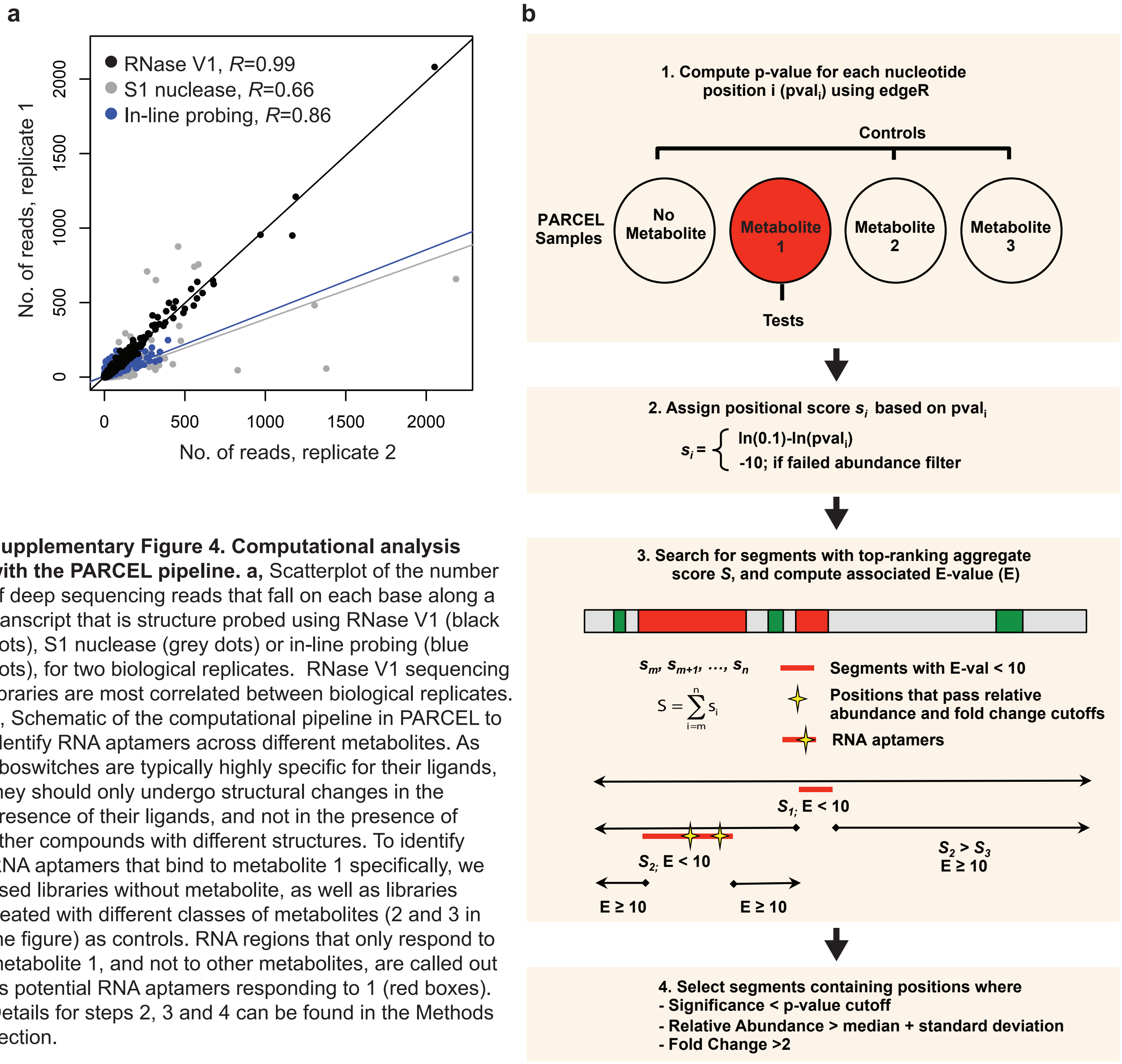

Supplementary Figure 4. Computational analysis with the PARCEL pipeline. a, Scatterplot of the number of deep sequencing reads that fall on each base along a transcript that is structure probed using RNase V1 (black dots), S1 nuclease (grey dots) or in-line probing (blue dots), for two biological replicates. RNase V1 sequencing libraries are most correlated between biological replicates. **b**, Schematic of the computational pipeline in PARCEL to identify RNA aptamers across different metabolites. As riboswitches are typically highly specific for their ligands, they should only undergo structural changes in the presence of their ligands, and not in the presence of other compounds with different structures. To identify RNA aptamers that bind to metabolite 1 specifically, we used libraries without metabolite, as well as libraries treated with different classes of metabolites (2 and 3 in the figure) as controls. RNA regions that only respond to metabolite 1, and not to other metabolites, are called out as potential RNA aptamers responding to 1 (red boxes). Details for steps 2, 3 and 4 can be found in the Methods section.

Supplementary Figure 5

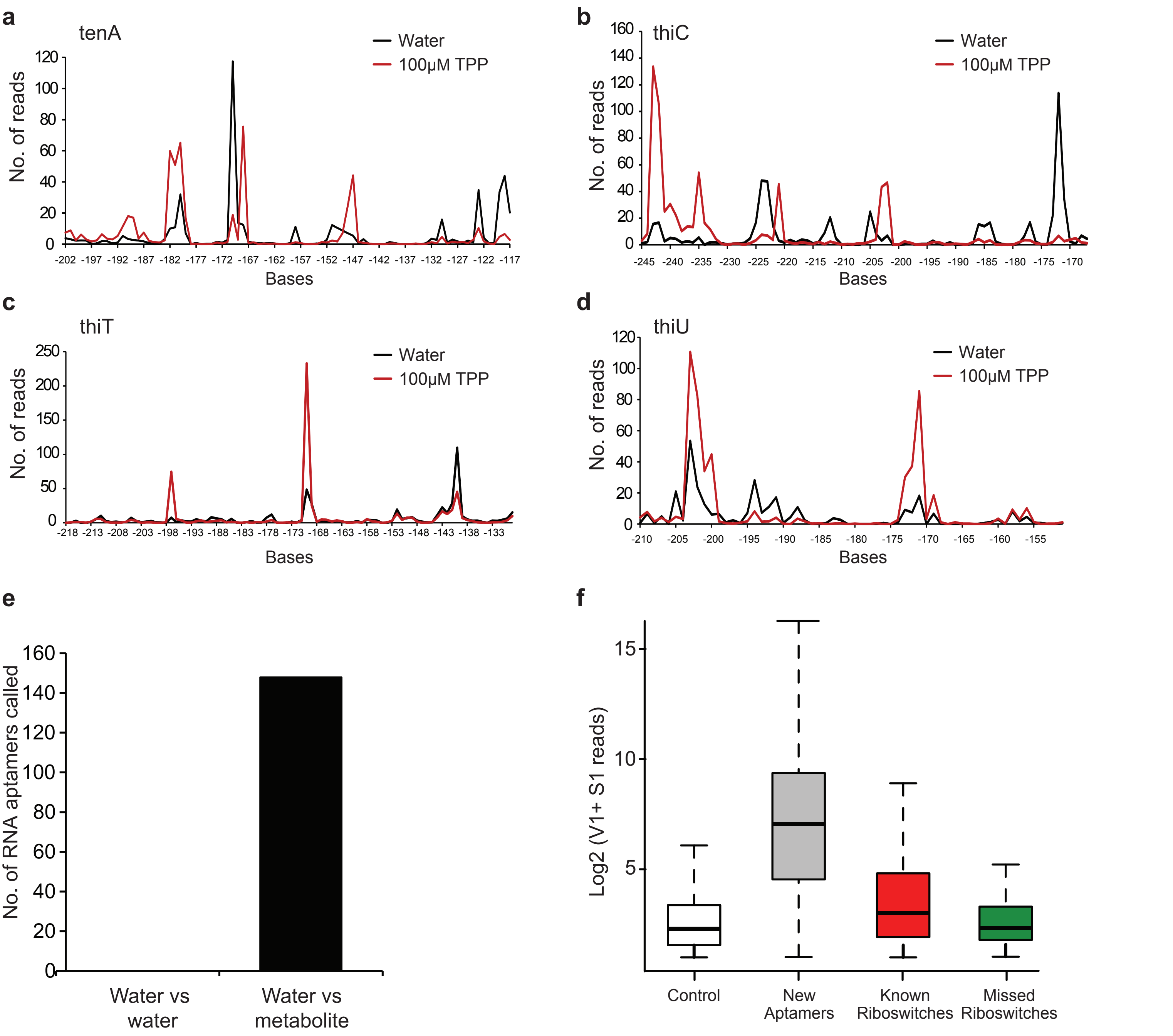

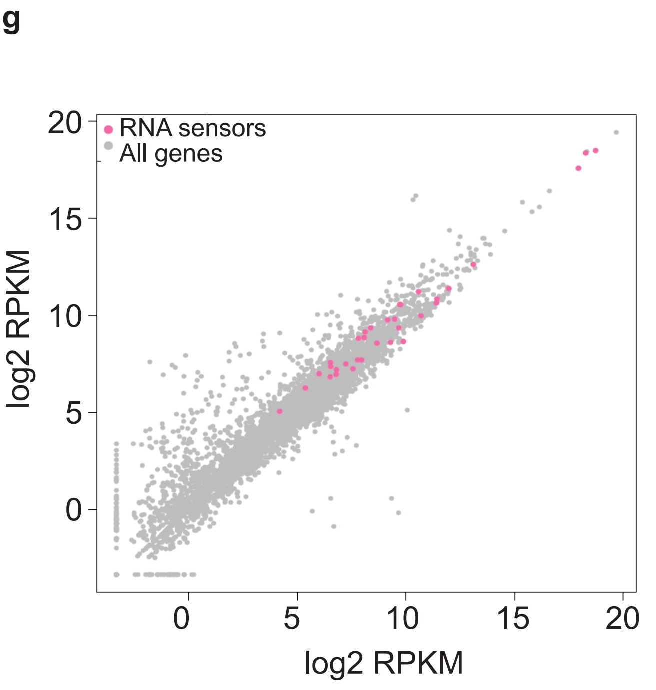

Supplementary Figure 5. PARCEL detects RNA aptamers sensitively and accurately. a-d, PARCEL sequencing reads for known *B. subtilis* TPP riboswitches tenA (**a**), thiC (**b**), thiT (**c**), and thiU (**d**) in the presence (red line) and absence (black line) of TPP[5]. **e**, PARCEL does not detect any RNA aptamers when RNAs under the same conditions are compared to each other (left). However, RNA aptamers are detected when metabolite-treated RNAs are compared to untreated RNAs (right), suggesting that there is very little noise in PARCEL data. **f,** Known riboswitches that fail to be detected by PARCEL reside in nuclease-inaccessible or poorly expressed regions in the transcriptome. The Y-axis indicates the sum of double- and single-stranded reads in a region, which is a measure for accessibility to RNase V1 and S1 nuclease. Our newly identified RNA aptamers (grey box), as well as PARCEL-detected known riboswitches (red box) are more accessible and/or abundant than the known riboswitches that we failed to detect using PARCEL (green box). **g**, Distribution of the abundance of new RNA aptamers (pink dots) and all transcripts (grey dots). X- and Y-axis represent log2(Reads per kilobase per million-RPKM) in two replicates.

Supplementary Figure 6

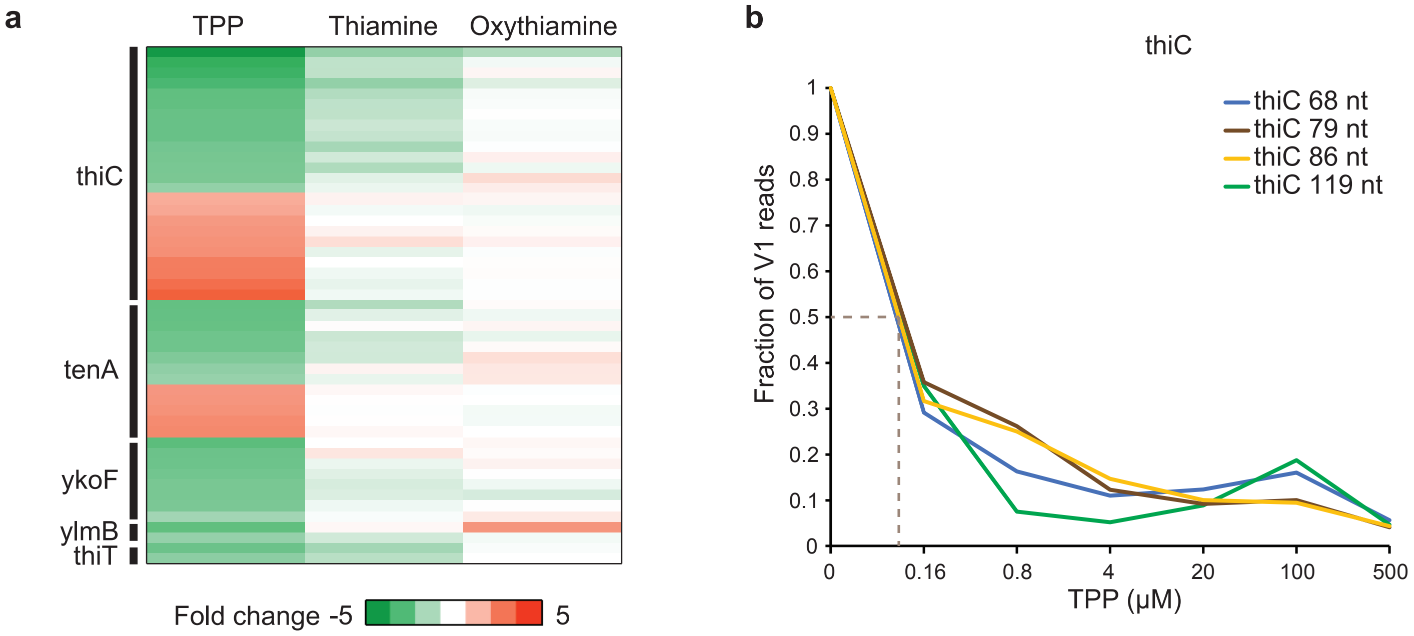

Supplementary Figure 6. PARCEL is quantitative in its measurements. a, PARCEL data for all five TPP riboswitches in *B. subtilis*. Each row indicates a position along an RNA that showed structural change. Red and green bars indicate increase or decrease in structuredness in the presence of metabolite respectively. All five TPP riboswitches exhibit the strongest structure change in the presence of TPP, followed by thiamine and oxythiamine. **b**, Plot showing how bases in the thiC riboswitch change structure (Y-axis: number of V1 reads at each base, normalized to sequencing depth) under increasing concentrations of TPP (X-axis). The grey dotted line indicates the K_D (110 nM) at half maximal point of structural change.

Supplementary Figure 7

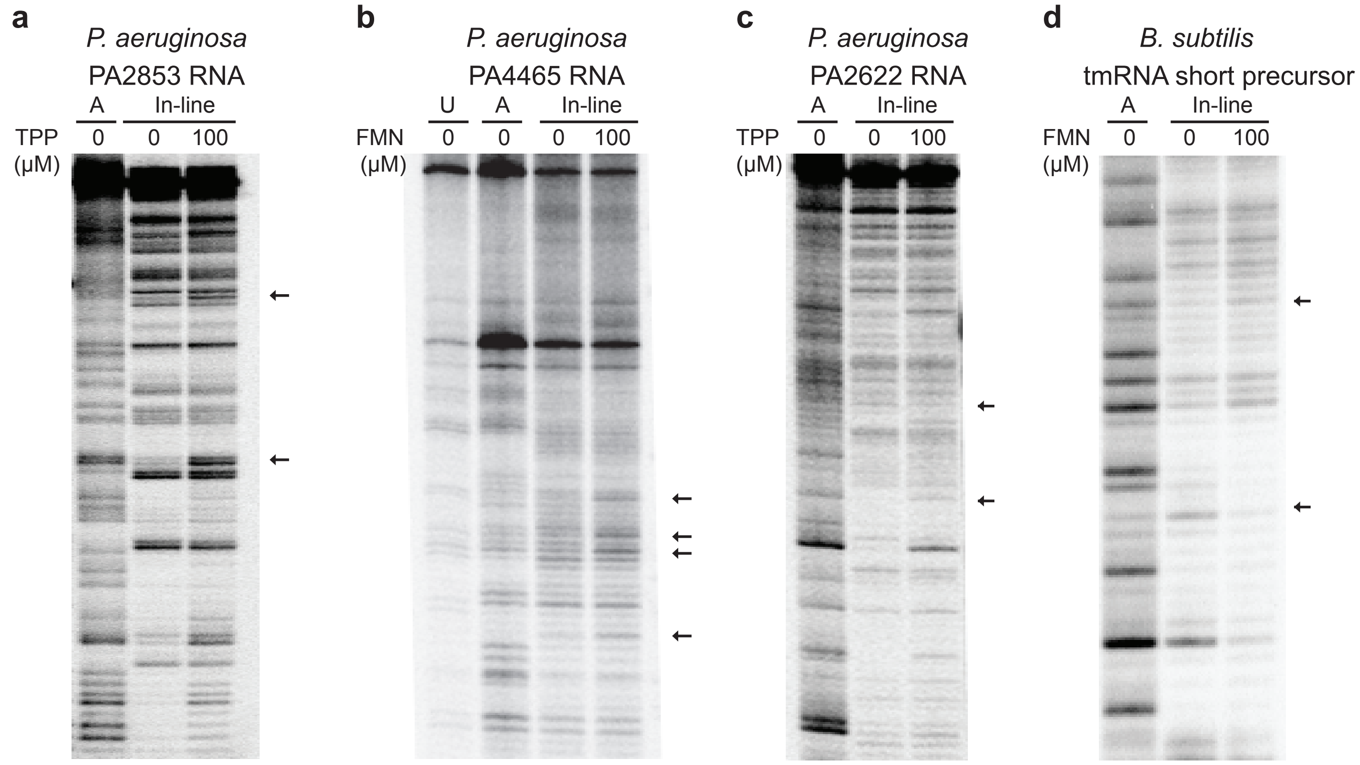

Supplementary Figure 7. Validation of new bacterial aptamers by in-line probing. a-d, Gel images of in-line probing performed on cloned and *in-vitro* transcribed TPP-responsive (**a, c**) and FMN-responsive (**b, d**) RNA aptamers found in *P. aeruginosa* and *B. subtilis* using PARCEL. Black arrows indicate regions of structural change in the presence of metabolite. Also shown are A ladder (A lane) and untreated full length RNA (U lane).

Supplementary Figure 8

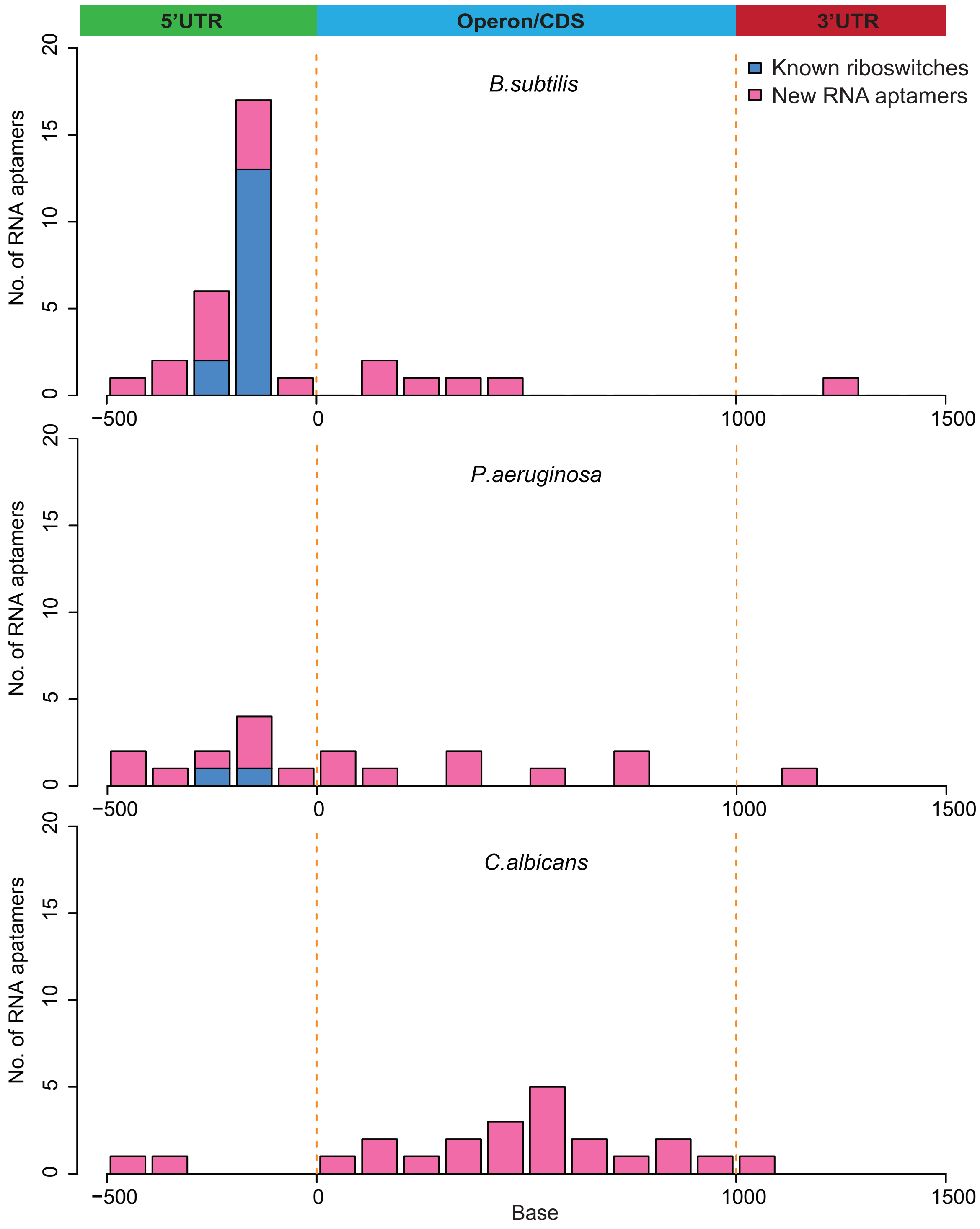

Supplementary Figure 8. Genomic locations of new RNA aptamers. Distribution of detected RNA aptamers upstream, downstream and within operons in *B. subtilis* (top), *P. aeruginosa* (middle), and in 5' UTRs, CDSs, and 3' UTRs in *C. albicans* (bottom). Y-axis indicates the number of aptamers that fall within each location. X-axis indicates the bases along the transcript. The blue and pink bars indicate the distribution of known riboswitches and new RNA aptamers respectively.

Supplementary Figure 9

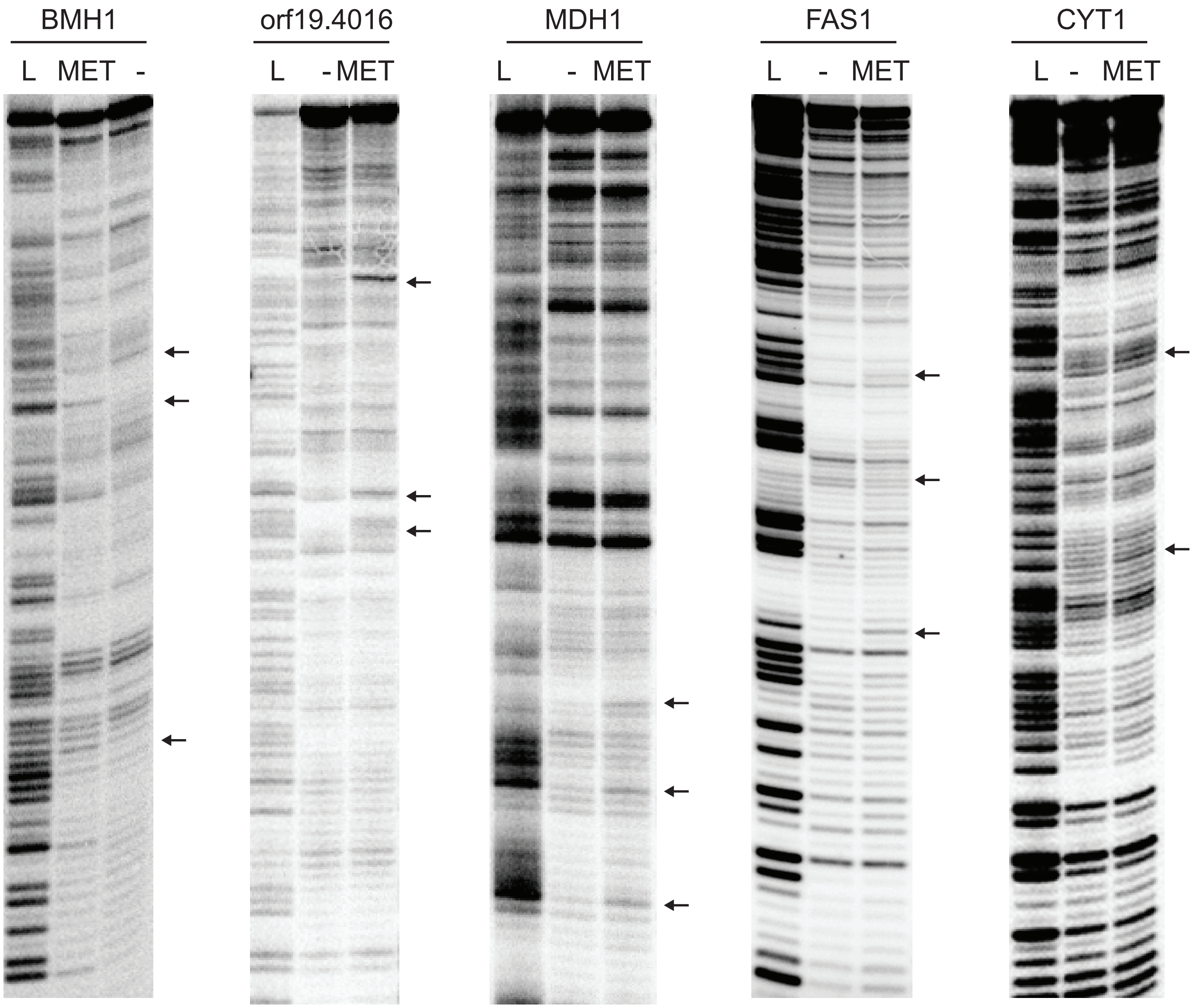

Supplementary Figure 9. Validation of *Candida albicans* RNA aptamers by in-line probing. Gel images of in-line probing reactions performed on cloned and *in-vitro* transcribed RNA aptamers found in *C. albicans* by PARCEL. BMH1 RNA was structured probed in the presence (lane 2) and absence (lane 3) of a pool of five metabolites (SAM, glycine, lysine, FMN and AdoCbl, each 100 μM). orf19.4016, MDH1, FAS1, CYT1 RNAs are structured probed in the presence (lane 3) and absence (lane 2) of a pool of five metabolites (SAM, glycine, lysine, FMN and AdoCbl, each 100 μM). The A ladder is also shown (lane 1). Black arrows indicate regions of structure change in the presence of metabolites.

Supplementary Figure 10

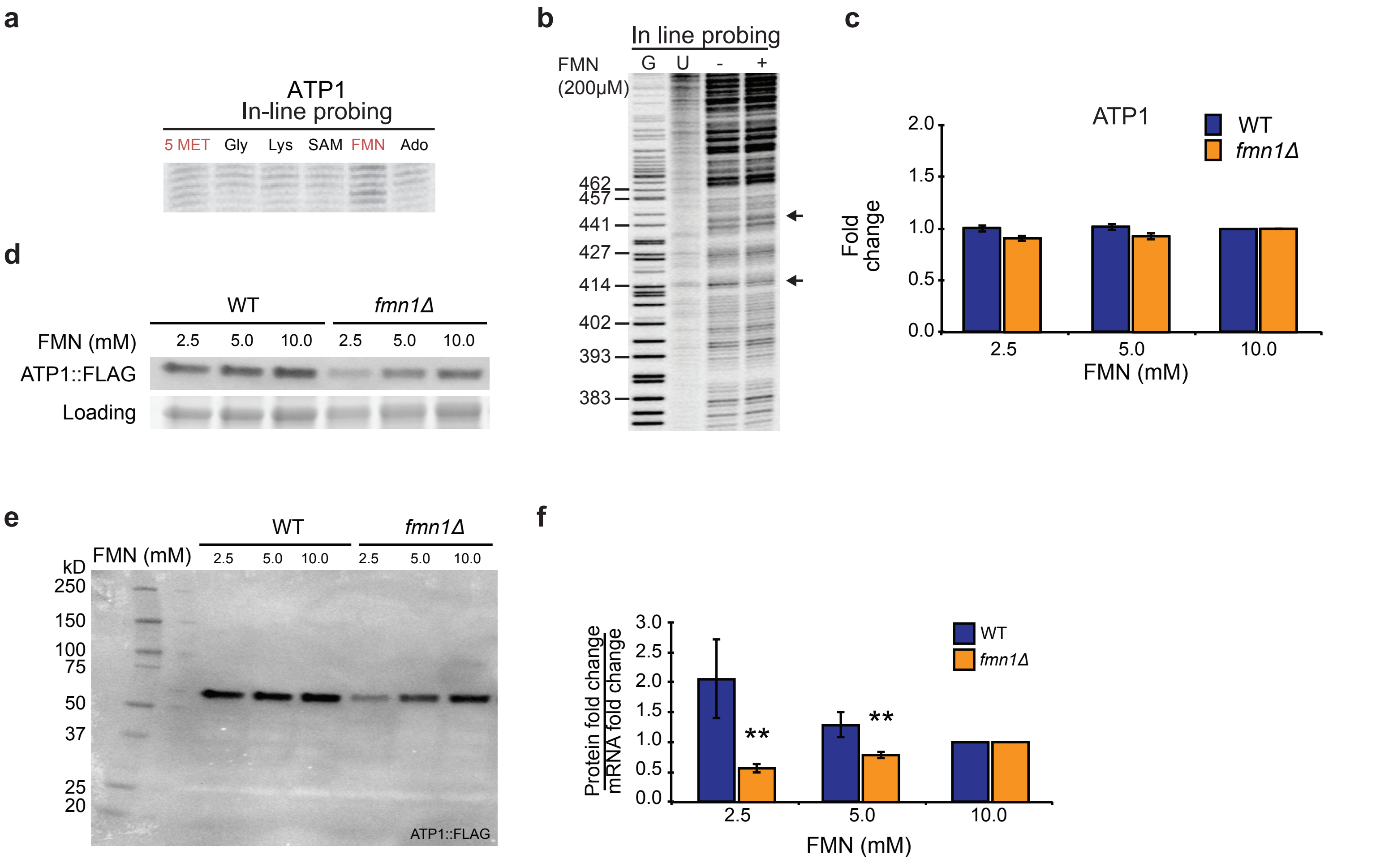

Supplementary Figure 10. Validation of *C. albicans* RNA sensor, ATP1. a, Gel images showing In-line probing of ATP1 mRNA in the presence of all 5 metabolites as a pool (Lane 1, 5 MET), 100μM glycine (Lane 2, gly),100μM lysine (Lane 3, lys), 100μM SAM (Lane 4), 100μM FMN (Lane 5), and 100μM Adocbl (Lane 6, Ado). **b**, Gel image of in-line probing reactions performed with *in vitro* transcribed and radiolabelled ATP1 RNA in the presence (lane 4) and absence (lane 3) of 200μM FMN. Also shown are the uncut RNA (lane 2) and G ladder (lane 1). Black arrows indicate regions of structure changes. **c**, Average normalized relative fold change (fc) levels of ATP1 mRNA (y-axis) were plotted against FMN concentrations (x-axis, mM). The blue and orange bars represent data from ATP1::FLAG knock-in strains with wildtype (WT) and deletion (*fmn1*Δ) backgrounds, respectively. The normalized relative fc levels were determined by first normalizing to actin (ACT1), and then to respective strain cultured at 10 mM FMN (fc = 1). The average and standard error (+/- error bar) were calculated from seven experiments. **d**, **e**, A representative Western blot showing ATP1::FLAG (top) and loading (bottom) protein levels in ATP1::FLAG knock-in strains with WT (left) and *fmn1*Δ (right) backgrounds cultured at different FMN concentrations (mM). Using t-test (n=7), significant *p*-values of 0.0003 and 0.001 (for 2.5 and 5.0 mM against 10.0 mM respectively) were determined for *fmn1*Δ, but not WT (*p*-values of 0.2 and 0.3). **f,** Average normalized (relative protein fc / relative mRNA fc) levels of ATP1::FLAG (y-axis) were plotted against FMN concentrations (x-axis, mM). The blue and orange bars represent data from ATP1::FLAG knock-in strains with WT and *fmn1*Δ backgrounds respectively. The relative protein fold changes were determined by normalizing to loading (non-specific band), whereas relative mRNA fold-changes were determined by normalizing to actin (ACT1). These values were then normalized to respective strain cultured at 10 mM FMN (fc = 1). The average and standard error (+/- error bar) were calculated from seven experiments. ** indicate significant *p*-values <0.01 (0.0006 and 0.004 for 2.5 mM and 5.0 mM respectively), as determined by t-test. WT *p*-values were 0.2 for both 2.5 mM and 5.0 mM FMN.

Supplementary Figure 11

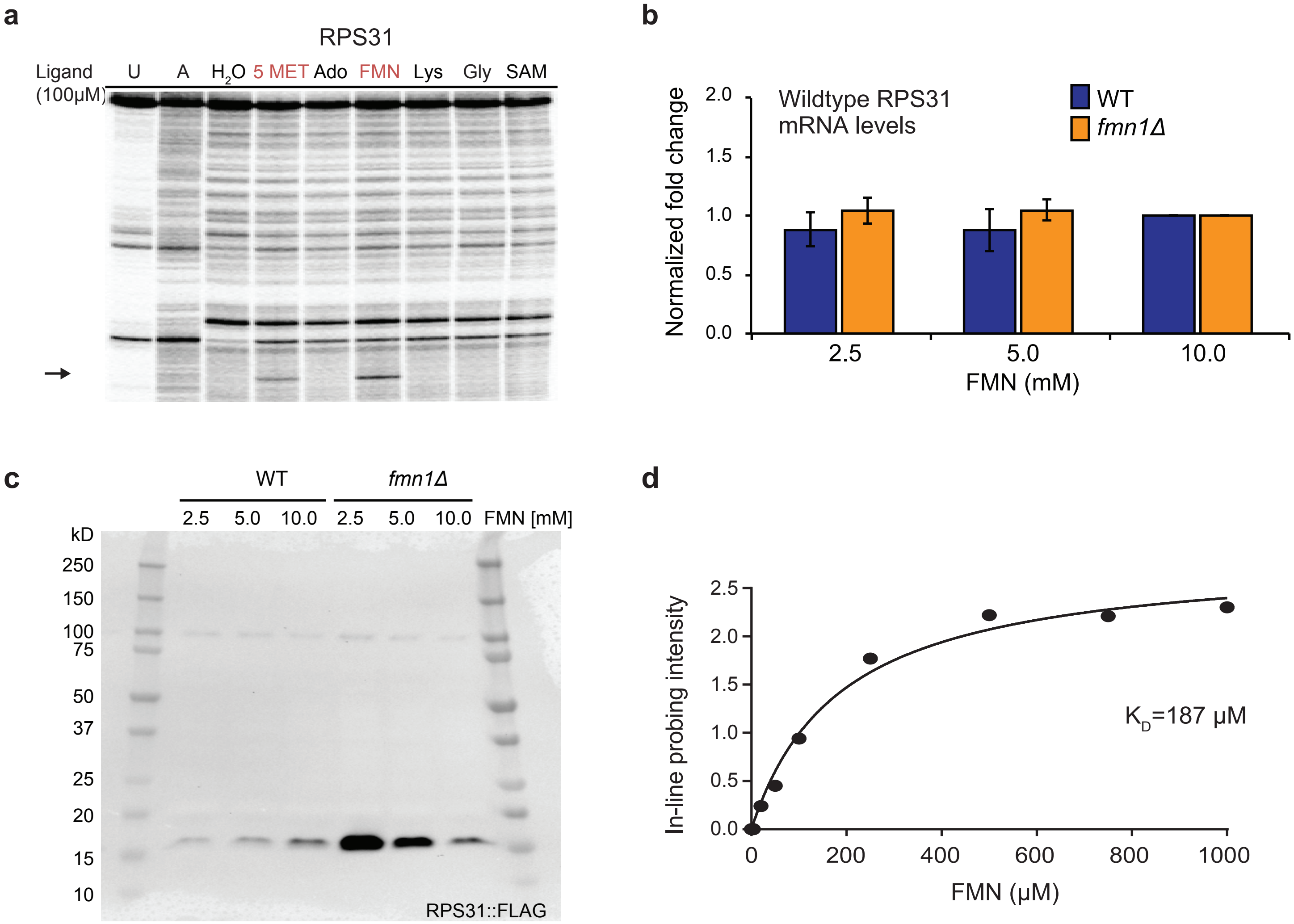

Supplementary Figure 11. Validation of *C. albicans* RNA sensor, RPS31. a, In-line probing of RPS31 RNA using a radiolabeled primer in the absence (Water, lane 3) and presence of all 5 metabolites as a pool (Lane 4, 5 MET), Adocbl (Lane 5, Ado), FMN (Lane 6), lysine (Lane 7, lys), glycine (Lane 8, gly), and SAM (Lane 9). Also shown are uncut RNA (lane 1) and A ladder (lane 2). The black arrow indicates a base that changes structure in the presence of FMN. **b**, Average normalized relative fold change (fc) levels of RPS31 mRNA (y-axis) were plotted against FMN concentrations (x-axis, mM). The blue and orange bars represent data from RPS31::FLAG knock-in strains with wildtype (WT) and deletion (*fmn1*Δ) backgrounds respectively. The normalized relative fc levels were determined by first normalizing to actin (ACT1), and then to respective strain cultured at 10 mM FMN (fc = 1). The average and standard error (+/- error bar) were calculated from eight experiments. **c**, Uncropped western blot image of Figure 4e, showing RPS31 protein levels change under different concentrations of FMN. **d**, Plot showing the intensity of in-line probing band with increasing concentration of FMN. The K_D of binding is estimated to be 187 μM based on a best-fit curve using non-linear regression. We used the parameters for single site binding to saturation in the program Prism.

Supplementary Figure 12

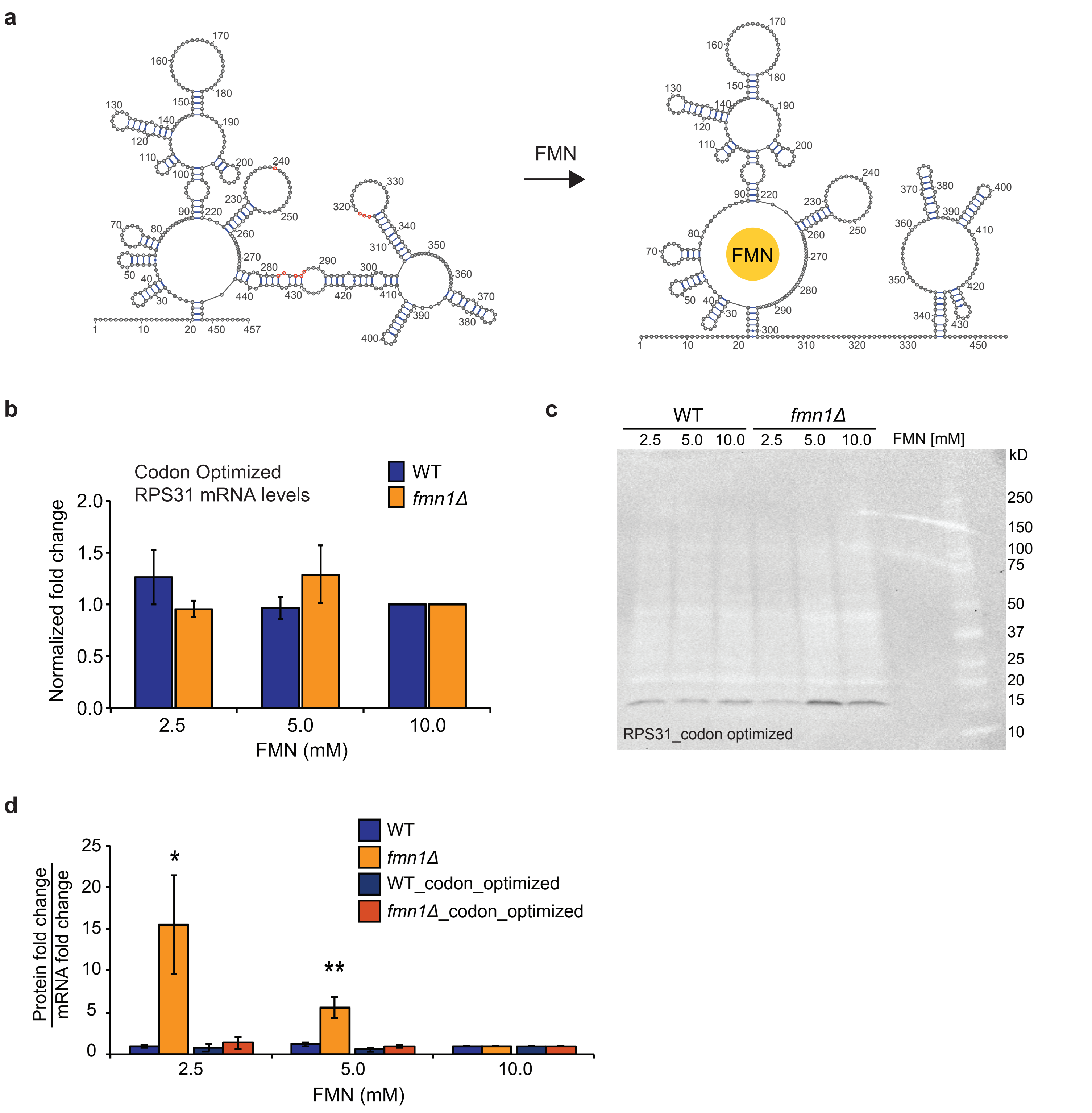

Supplementary Figure 12. a, Secondary structure models of the free (left) and FMN bound (right) RPS31 mRNA using RNase V1, S1, SHAPE and in-line probing signals as constraints in the program RNAfold. The red bases indicate positions that became more single-stranded, upon ligand binding, in our footprinting analysis. **b**, Average normalized relative fold change (fc) levels of codon-optimized RPS31 (co_RPS31) mRNA (y-axis) were plotted against FMN concentrations (x-axis, mM). The blue and orange bars represent data from co_RPS31::FLAG knock-in strains with WT and *fmn1*Δ backgrounds respectively. The normalized relative fc levels were determined by first normalizing to actin (ACT1), and then to respective strain cultured at 10 mM FMN (fc = 1). The average and standard error (+/- error bar) were calculated from three experiments. **c**, Uncropped western blot image of codon optimized RPS31::FLAG in the presence of different concentrations of FMN (**Figure 4h**). **d**, Average normalized (relative protein fc / relative mRNA fc) levels of RPS31::FLAG (y-axis) were plotted against FMN concentrations (x-axis, mM). The blue and orange bars represent data from RPS31::FLAG knock-in strains with WT and *fmn1*Δ backgrounds respectively, while the dark blue and red bars represent data from co_RPS31::FLAG knock-in strains with WT and *fmn1*Δ backgrounds respectively. The relative protein fold changes were determined by normalizing to loading (non-specific band), whereas relative mRNA fold changes were determined by normalizing to actin (ACT1). These values were then normalized to respective strain cultured at 10 mM FMN (fc = 1). The average and standard error (+/- error bar) were calculated from at least three experiments. * and ** indicate significant *p*-values <0.05 (0.04 for 2.5 mM FMN) and <0.01 (0.009 for 5.0 mM FMN) respectively, as determined by t-test. *P*-values for co_RPS31::FLAG knock-in strains were not significant (between 0.2 and 0.8).

Supplementary Table1. Mapping Statistics of PARCEL libraries

Species	Condition	Replicates	Sequenced	With Adapter	After adapter Trimmed	Mapped	Uniquely Mapped	Mappability (%)
B.subtilis	fad	rep1	10833015	8322162	7356648	5231848	4983216	71.12
B.subtilis	fad	rep2	10447817	8597632	6549922	4781118	4545679	73.00
B.subtilis	sam	rep1	11430669	9037182	7814019	5464075	5200934	69.93
B.subtilis	sam	rep2	10521498	8514137	6504582	4883165	4656615	75.07
B.subtilis	water	rep1	10871016	6912432	7820288	5546480	5302016	70.92
B.subtilis	water	rep2	10598006	7387124	7170237	5213999	4961003	72.72
B.subtilis	fmn	rep1	3508089	1956891	2698306	2344049	2264024	86.87
B.subtilis	fmn	rep2	2799587	1598995	1773163	1531226	1484117	86.36
B.subtilis	tpp	rep1	9128018	6101418	5966587	5397650	5203978	90.46
B.subtilis	tpp	rep2	5942019	3608533	3269960	3012528	2944283	92.13
B.subtilis	water	rep1	9566093	6047195	5749303	5228163	5047189	90.94
B.subtilis	water	rep2	7623405	4618343	5010175	4547478	4403194	90.76
C.albicans	pool of metabolites	rep1	95399843	44315134	83524057	3.4E+07	32698144	41.02
C.albicans	pool of metabolites	rep2	105480861	51841834	95012910	4.3E+07	40843361	45.03
C.albicans	tpp	rep1	118986513	44269951	108190741	4.8E+07	45972371	44.48
C.albicans	tpp	rep2	136255424	71921380	116472194	5.5E+07	52046672	46.83
C.albicans	water	rep1	99743550	45756969	90135506	4.1E+07	38949194	45.29
C.albicans	water	rep2	99413312	47944576	79783124	3.9E+07	37369447	49.41
P.aeruginosa	fmn	rep1	10790637	6836390	6457721	5096640	4963238	78.92
P.aeruginosa	fmn	rep2	11487412	7259000	6984632	5663270	5508889	81.08
P.aeruginosa	tpp	rep1	9295428	4251067	5997412	5064108	4988672	84.44
P.aeruginosa	tpp	rep2	9810404	6698194	4544656	3726769	3649732	82.00
P.aeruginosa	water	rep1	10722458	5048775	8642005	7227862	7055499	83.64
P.aeruginosa	water	rep2	11325037	4958754	7919815	6588814	6360793	83.19

Supplementary Figure 2. List of RNA aptamers found in prokaryotes

Species	Ligand	Genomic Region	Locus Name	Biotype	Closest Gene	Region Type	Known	Fold change	E-value
B.subtilis	sam	NC_000964:1180679-1180906	BSU_misc_RNA_12	processed_transcript	BSU11010	5UTR	Yes	9.11	0.00E+00
B.subtilis	fmn	NC_000964:1180711-1180734	BSU_misc_RNA_12	processed_transcript	BSU11010	5UTR	No	2.01	1.34E-24
B.subtilis	fmn	NC_000964:1219266-1219277	BSU_misc_RNA_13	processed_transcript	BSU11420	5UTR	No	2.17	2.97E-03
B.subtilis	tpp	NC_000964:1242243-1242372	BSU_misc_RNA_14	processed_transcript	BSU11650	5UTR	Yes	16.50	1.39E-38
B.subtilis	sam	NC_000964:1258281-1258426	BSU_misc_RNA_15	processed_transcript	BSU11870	5UTR	Yes	65.83	9.97E-06
B.subtilis	sam	NC_000964:126665-126666	rpoC	protein_coding	BSU01080	CDS	No	2.24	1.68E+00
B.subtilis	fmn	NC_000964:133423-133477	tufA	protein_coding	BSU01130	CDS	No	2.54	2.77E-45
B.subtilis	fmn	NC_000964:137444-137449	rplB	protein_coding	BSU01190	CDS	No	3.87	2.00E-02
B.subtilis	sam	NC_000964:1385725-1386012	BSU_misc_RNA_18	processed_transcript	BSU13180	5UTR	Yes	80.50	8.07E-138
B.subtilis	tpp	NC_000964:1391738-1391852	BSU_misc_RNA_19	processed_transcript	BSU13240	5UTR	Yes	24.15	9.15E-63
B.subtilis	fmn	NC_000964:139912-139942	rplP	protein_coding	BSU01240	5UTR	No	3.65	9.86E-14
B.subtilis	sam	NC_000964:1424526-1424710	BSU_misc_RNA_20	processed_transcript	BSU13560	5UTR	Yes	19.76	5.85E-28
B.subtilis	sam	NC_000964:1426865-1426978	BSU_misc_RNA_21	processed_transcript	BSU13590	5UTR	Yes	66.60	1.18E-13
B.subtilis	fmn	NC_000964:1459076-1459081	ptsG	protein_coding	BSU13900	5UTR	No	2.01	5.23E-02
B.subtilis	sam	NC_000964:1524309-1524316	rnjA	protein_coding	BSU14530	CDS	No	7.49	6.11E-02
B.subtilis	fmn	NC_000964:1673762-1673768	rpsP	protein_coding	BSU16000	5UTR	No	5.05	1.02E+00
B.subtilis	sam	NC_000964:2025159-2025252	BSU_misc_RNA_31	processed_transcript	BSU18560	5UTR	Yes	23.44	1.80E+00
B.subtilis	fmn	NC_000964:2070018-2070025	NA	NA	BSU18980	3UTR	No	3.09	3.12E-02
B.subtilis	sam	NC_000964:2394851-2394856	rpsA	protein_coding	BSU22870	5UTR	No	2.25	9.09E+00
B.subtilis	fmn	NC_000964:2410608-2410899	ypzE	protein_coding	BSU23050	5UTR	Yes	163.95	9.53E-08
B.subtilis	fmn	NC_000964:2431328-2431560	BSU_misc_RNA_36	processed_transcript	BSU23280	5UTR	Yes	8.95	1.53E-52
B.subtilis	sam	NC_000964:2431541-2431548	BSU_misc_RNA_36	processed_transcript	BSU23280	5UTR	No	2.85	1.50E-02
B.subtilis	fmn	NC_000964:26464-26619	scr	processed_transcript	BSU00190	5UTR	No	2.38	2.14E-218
B.subtilis	fmn	NC_000964:2814509-2814515	BSU_misc_RNA_41	processed_transcript	BSU27540	5UTR	No	9.11	5.51E-03
B.subtilis	sam	NC_000964:3129194-3129334	BSU_misc_RNA_50	processed_transcript	BSU30550	5UTR	Yes	8.01	1.41E-12
B.subtilis	tpp	NC_000964:3179105-3179208	BSU_misc_RNA_51	processed_transcript	BSU30990	5UTR	Yes	6.77	1.76E-03
B.subtilis	sam	NC_000964:3358682-3358695	sufD	protein_coding	BSU32690	5UTR	No	4.51	6.99E-05
B.subtilis	sam	NC_000964:3364396-3364504	BSU_misc_RNA_53	processed_transcript	BSU32750	5UTR	Yes	5.54	9.88E-02
B.subtilis	fmn	NC_000964:3450741-3451095	ssrA	tmRNA	BSU33600	5UTR	No	9.00	7.72E-66
B.subtilis	tpp	NC_000964:3450955-3450985	ssrA	tmRNA	BSU33590	5UTR	No	2.33	1.32E-18
B.subtilis	sam	NC_000964:3999147-3999274	BSU_misc_RNA_62	processed_transcript	BSU38960	5UTR	Yes	22.07	2.81E-03
B.subtilis	sam	NC_000964:4156515-4156516	purA	protein_coding	BSU40420	CDS	No	2.62	1.20E+00
B.subtilis	tpp	NC_000964:955647-955764	BSU_misc_RNA_11	processed_transcript	BSU08790	5UTR	Yes	24.51	5.75E-113
P.aeruginosa	tpp	NC_002516:2965124-2965125	NA	NA	PA2622	5UTR	No	4.13	3.45E+00
P.aeruginosa	tpp	NC_002516:2966734-2966740	icd	protein_coding	PA2623	CDS	No	7.76	3.56E-02
P.aeruginosa	tpp	NC_002516:3119316-3119328	NA	NA	PA2758	3UTR	No	5.09	4.30E-18

P.aeruginosa	tpp	NC_002516:3207100-3207119	oprI	protein_coding	PA2853	CDS	No	2.23	2.06E-07
P.aeruginosa	fmn	NC_002516:4536676-4536861	NA	NA	PA4055	5UTR	Yes	31.58	3.91E-24
P.aeruginosa	tpp	NC_002516:4754571-4754577	rpoA	protein_coding	PA4237	5UTR	No	4.19	4.39E-02
P.aeruginosa	tpp	NC_002516:4770822-4770823	fusA1	protein_coding	PA4266	CDS	No	4.60	3.67E+00
P.aeruginosa	tpp	NC_002516:4782402-4782409	rplA	protein_coding	PA4273	CDS	No	19.61	2.19E-04
P.aeruginosa	tpp	NC_002516:4785861-4785864	PA4277.3	tRNA	PA4277	5UTR	No	2.31	1.25E-05
P.aeruginosa	tpp	NC_002516:4893916-4893928	sodB	protein_coding	PA4366	CDS	No	5.42	3.72E-05
P.aeruginosa	tpp	NC_002516:4917085-4917092	groEL	protein_coding	PA4385	CDS	No	8.77	7.35E-01
P.aeruginosa	tpp	NC_002516:4956479-4956486	rnpB	ncRNA	PA4421	5UTR	No	2.31	3.63E-03
P.aeruginosa	fmn	NC_002516:4994766-4994771	ptsN	protein_coding	PA4465	5UTR	No	8.34	3.12E-01
P.aeruginosa	tpp	NC_002516:5322557-5322560	PA4738	protein_coding	PA4738	CDS	No	132.46	1.73E-02
P.aeruginosa	tpp	NC_002516:5325732-5325743	rpsO	protein_coding	PA4740	5UTR	No	2.79	3.32E-04
P.aeruginosa	tpp	NC_002516:5583734-5583879	NA	NA	PA4973	5UTR	Yes	44.11	7.23E-30
P.aeruginosa	tpp	NC_002516:5623233-5623236	PA5005	protein_coding	PA5004	5UTR	No	15.12	4.10E+00
P.aeruginosa	tpp	NC_002516:5884330-5884354	ssrS	ncRNA	PA5228	5UTR	No	2.23	2.54E-08
P.aeruginosa	tpp	NC_002516:932351-932352	PA0853	protein_coding	PA0853	CDS	No	2.82	1.56E+00

Supplementary Table 3. List of RNA aptamers found in *C. albicans*

Species	Region	Locus Name	Closest Gene	Region Type	Known	Fold Change	E-value
C.albicans	Ca21chr1:1846664-1846715	GCV2	orf19.385	CDS	No	2.18	4.89E+00
C.albicans	Ca21chr1:1883720-1883770	GCD6	orf19.407	CDS	No	5.18	6.21E+00
C.albicans	Ca21chr1:2634070-2634125	SER33	orf19.5263	5UTR	No	2.78	6.30E-01
C.albicans	Ca21chr1:506778-506829	GSC1	orf19.2929	CDS	No	2.05	1.20E-01
C.albicans	Ca21chr1:583654-583705	TIF34	orf19.2967	CDS	No	4.22	7.44E-02
C.albicans	Ca21chr1:675684-675737	BMH1	orf19.3014	CDS	No	2.32	5.35E-07
C.albicans	Ca21chr1:677952-678010	ARX1	orf19.3015	CDS	No	3.55	1.58E-01
C.albicans	Ca21chr2:1033417-1033493	CYT1	orf19.3527	CDS	No	2.08	5.94E-04
C.albicans	Ca21chr2:1508031-1508084	SSC1	orf19.1896	CDS	No	2.49	4.14E-08
C.albicans	Ca21chr2:1512787-1512837		orf19.1891	CDS	No	5.47	2.76E-02
C.albicans	Ca21chr2:876857-876913	orf19.804.1	orf19.804.1	CDS	No	2.73	2.48E-07
C.albicans	Ca21chr3:1096972-1097099	RPL18	orf19.5982	CDS	No	2.68	1.43E-80
C.albicans	Ca21chr4:1585877-1586030	UBI3	orf19.3087	5UTR/CDS	No	3.19	1.46E-40
C.albicans	Ca21chr4:371006-371072	MDH1-1	orf19.4602	CDS	No	2.07	7.23E-17
C.albicans	Ca21chr5:1146016-1146096	orf19.4016	orf19.4016	CDS	No	2.72	3.33E-05
C.albicans	Ca21chr5:24826-25000	FAS1	orf19.979	CDS	No	2.57	5.61E-16
C.albicans	Ca21chr5:26160-26212	FAS1	orf19.979	CDS	No	2.09	1.61E+00
C.albicans	Ca21chr6:133205-133257	PGK1	orf19.3651	CDS	No	2.83	8.73E-01
C.albicans	Ca21chrR:1387851-1387901	HSP60	orf19.717	CDS	No	2.36	3.82E-03
C.albicans	Ca21chrR:1508821-1508871	MIS11	orf19.2364	CDS	No	2.50	7.46E+00
C.albicans	Ca21chrR:1881062-1881112	tE(UUC)7	orf19.6449	3UTR	No	3.04	4.09E-01
C.albicans	Ca21chrR:1946197-1946248	TUB1	orf19.7308	CDS	No	2.19	7.62E+00
C.albicans	Ca21chrR:384669-384720	CDC60	orf19.2560	CDS	No	2.43	3.02E+00
C.albicans	Ca21chr1:950442-950479	ATP1	orf19.6854	CDS	No	4.42	1.48E-09

Supplementary References

1. Das, R., Laederach, A., Pearlman, S. M., Herschlag, D. & Altman, R. B. SAFA: semi-automated footprinting analysis software for high-throughput quantification of nucleic acid footprinting experiments. *RNA* **11**, 344-354 (2005).

2. Winkler, W. C., Nahvi, A., Sudarsan, N., Barrick, J. E. & Breaker, R. R. An mRNA structure that controls gene expression by binding S-adenosylmethionine. *Nat. Struct. Biol.* **10**, 701-707 (2003).

3. Regulski, E. E. & Breaker, R. R. In-line probing analysis of riboswitches. *Methods Mol. Biol.* **419**, 53-67 (2008).

4. Sudarsan, N., Wickiser, J. K., Nakamura, S., Ebert, M. S. & Breaker, R. R. An mRNA structure in bacteria that controls gene expression by binding lysine. *Genes Dev.* **17**, 2688-2697 (2003).

5. Novichkov, P. S. *et al*. RegPrecise 3.0--a resource for genome-scale exploration of transcriptional regulation in bacteria. *BMC Genomics* **14**, 745-2164-14-745 (2013).
